# Naturalistic randomized controlled trial demonstrating effectiveness of Text4Hope in supporting male population mental health during the COVID-19 pandemic

**DOI:** 10.3389/fpubh.2022.1002288

**Published:** 2022-09-26

**Authors:** Reham Shalaby, Belinda Agyapong, Wesley Vuong, Marianne Hrabok, April Gusnowski, Shireen Surood, Andrew J. Greenshaw, Vincent I. O. Agyapong

**Affiliations:** ^1^Department of Psychiatry, Faculty of Medicine and Dentistry, University of Alberta, Edmonton, AB, Canada; ^2^Addiction and Mental Health, Alberta Health Services, Edmonton, AB, Canada; ^3^Department of Psychiatry, Faculty of Medicine and Dentistry, University of British Columbia, Vancouver, BC, Canada; ^4^Department of Psychiatry, Faculty of Medicine and Dentistry, Dalhousie University, Halifax, NS, Canada

**Keywords:** males, Text4Hope, naturalistic randomized controlled trial, mental health, COVID-19, texting services

## Abstract

**Background:**

Mental illness is not uncommon among males. It is estimated that males are more likely to die by suicide, become dependent on alcohol, report frequent drug use, and be dissatisfied with their life, compared to women. In this study, we assessed the potential to offer support to this population using Text4Hope, a texting mental health service.

**Methods:**

The study was a naturalistic randomized controlled trial comparing two populations of Text4Hope male subscribers; an intervention group (IG, Text4Hope subscribers who received once-daily supportive text messages for 6 weeks) and a control group (CG, Text4Hope subscribers who joined the program in the same time frame but were yet to receive text messages). Inferential statistics were used to compare the severity and the prevalence of the likely stress, anxiety, and depression, between the two groups, using the Perceived Stress Scale (PSS-10), the Generalized Anxiety Disorder 7-item (GAD-7), and the Patient Health Questionnaire-9 (PHQ-9), and defined the Composite Mental Health (CMH) score as the sum of these three. *T*-test, Chi-squared association, and binary logistic regression analyses were applied.

**Results:**

There were 286 male subscribers to Text4Hope. The majority were above 40 years, white, employed, had postsecondary education, were in a relationship, and owned a home. Mean scores of PSS-10, GAD-7, and PHQ-9 scales and the CMH were significantly higher for the CG compared to the IG, 11.4, 28.8, 25.8, and 18.7%, respectively. Similarly, a statistically significantly lower prevalence in IG, compared to the CG, on likely MDD (58.15 vs. 37.4%) and likely GAD (50 vs. 30.8%), with a small effect size. The IG was a significant predictor for lower odds of both likely MDD and likely GAD while controlling for sociodemographic characteristics.

**Conclusions:**

The Text4Hope service is an effective tool for mental health support for male subscribers during the COVID-19 pandemic. Compared to the males who didn't receive the service, those who received it were in better mental health conditions. Further effort is still needed to encourage males to participate in such online services that can help them receive adequate support, particularly during crisis times.

## Introduction

While mental illnesses affect both men and women, the latest studies report an increasing trend of mental illness among men (1, 2). It is estimated that three times as many men as women die by suicide, become dependent on alcohol, and report frequent drug use (3). Further, males are more likely to be less satisfied with their lives (3). The literature suggests that males tend to deny mental health concerns and do not seek professional support for their psychological problems. According to Ketterer et al. (4) more females often report depression and anxiety than males. However, anger and denial are usually greater in males. Contributing to the problem, the high levels of emotional stress experienced by men can be a risk factor for the predisposition to physical health adversities, including heart problems (4, 5).

Mental illness is common among males as much as females. According to the Canadian Mental Health Association, one in five people in Canada will personally experience a mental health problem or illness in any given year (6). The StatsCan Canadian Community Health Survey on Mental health and wellbeing found a comparable survey of mental health disorders and substance dependencies among men and women (10% of men compared to 11% of women) (1). According to the Centre for Disease Control and Prevention, men are often less likely to have received mental health treatment than women in the past year (7). This includes medications, counseling, or therapy. The same trend was also noted among the men who endure a severe mental illness (7).

The gender difference in mental illness represents a health disparity that cannot be overlooked. Men are less likely to get the appropriate mental health diagnosis when compared with females who experience the same symptoms or scores on diagnostic scales (8, 9). Such disparity is often reported among males, particularly young adolescents with depressive symptoms (10). Furthermore, the quite different framework of symptomatology of mental illness in males compared to females has added to the problem. From the literature, it was suggested that men might experience mental illness differently than females. For example, depression among males could manifest as irritability, aggression, violence, substance abuse, risky behavior, or somatic complaints (9, 11). Such symptoms are typically not included in the diagnostic criteria of the condition; therefore, they could be easily overlooked without obtaining a formal diagnosis (9, 11).

Several variables may contribute to experiencing mental illnesses among males. This includes genetics, social, and psychological factors. Unemployment, along with the sense of competition with the other gender, can adversely affect the feeling of power (1). Men are often less likely to seek help. Thus, the severe life-threatening conditions can distort their sense of strength and power, aggravating their depressed feeling (1).

In another context, the COVID-19 pandemic has impacted individuals' physical as well as mental health. According to the World health organization, in 2019, nearly a billion people were living with a mental disorder. In the first year of the pandemic alone, depression and anxiety went up by more than 25% (12). The detrimental impacts of the pandemic reflected in morbidity and mortality rates, changes in ways of living and other usual daily activities, along with the uncertainties associated with the pandemic, have mounted up to the experience of mental health adversities (13, 14). Physical and mental long-term sequelae among COVID-19 survivors are high among males (15). Whereas, depression and anxiety were more common among women, men were more likely to endure PTSD during the pandemic (16).

Additionally, male mental health is not highlighted enough in the literature, particularly during the pandemic. This is partly attributed to the nature of data which is often gathered *via* self-reported questionnaire surveys that typically attract more females than males. Previous publications often report more female service subscribers are more likely to receive the online service and report on their mental health using validated scales compared to their male counterparts (17–19). Uneven gender sampling may skew results or limit the ability to generalize to the broader population.

Text4Hope is a remotely delivered service stimulated by the pandemic to integrate technology-based mental health supports provided to the general population in Alberta, Canada, during the COVID-19 pandemic (20, 21). To solicit the mental illness and the potential support among the male population, this study was designed to focus on the assessment of the Text4Hope service outcome among only male subscribers who have received the service for 6 consecutive weeks, compared to the male subscribers who have not received the service, during the COVID-19 pandemic.

## Methodology

### Study design

This study focused on male subscribers of the Text4Hope service. The design represented a naturalistic randomized controlled trial, comparing two study populations of Text4Hope subscribers who identified as male. The first group was an intervention group (IG) who were subscribers that received once daily supportive text messages for 6 weeks and completed 6 week evaluation measures between April 26 and July 12, 2020. The second group was a control group (CG), who were subscribers who joined the program in the same time frame and completed baseline evaluation measures and did not receive any text messages yet.

The Research and Ethics Board approved the study protocol (21) of the University of Alberta (Pro00086163).

### Text4Hope

In the Text4Hope program (22), individuals self-subscribe to receive daily supportive SMS text messages for 3 months by texting the word “COVID19HOPE” to a short code number. Detailed information about the service was described in previous publications (23, 24). In brief, the messages were crafted within a cognitive behavioral framework, with content written by mental health professionals. The first message welcomed subscribers to the service and invited them to complete a baseline survey. At 6 weeks, subscribers were invited again *via* a text message link to complete another web-based follow-up survey.

### Data collection

Participation in the Text4Hope service was voluntary, and the completion of the associated online surveys was not pertinent to receiving supportive SMS text messages. Subscribers could opt out of the service at any time. The surveys captured various sociodemographic data provided as categorical factors, including age (<=40 y, >40 y), ethnicity (White, non-white), educational level (postsecondary education, high school diploma or less), employment status (employment, not employed), relationship status (in a relationship, not in a relationship), and housing status (own home, other). Clinical information was also collected, including various mental health self-reported symptoms, which represented the study outcome measures.

### Outcome measures

At baseline and 6 weeks, we collected clinical information on stress, anxiety, and depression based on validated screening scales for self-reported symptoms, including the Perceived Stress Scale (PSS; PSS score ≥ 14 indicates moderate or high stress) (25), the Generalized Anxiety Disorder 7-item (GAD-7) Scale [GAD-7 score ≥ 10 indicates likely generalized anxiety disorder (GAD)] (26), the Patient Health Questionnaire-9 [PHQ-9; a score ≥ 10 indicates possible major depressive disorder (MDD)] (27), and defined the Composite Mental Health (CMH) score as the sum of these three. These measures have been shown to have strong reliability and validity.

### Hypothesis

We hypothesized that male participants who have received the daily supportive text messages for 6 weeks (IG), when compared to Text4Hope subscribers who were yet to receive the intervention (CG), would have at least 25% lower scores on CMH score, PSS-10, PHQ-9, and GAD-7 scales, in addition to the respective prevalence for each of moderate/high stress, likely GAD, and likely MDD.

### Sample size considerations

To detect a 25% difference in mean CMH score between the IG and the CG, given a two-sided significance level α = 0.05 and a power of 80% (β = 0.2), we estimated a sample size of 62 per group would be sufficient.

### Statistical analysis

Data analysis was undertaken using SPSS for Windows version 25 (IBM Corporation) (28). We examined the difference between the mean scores of PSS-10, GAD-7, PHQ-9 scales, and CMH scores between the IC and CG using independent *t*-tests. In addition, we examined the prevalence of moderate to high stress, likely GAD, and likely MDD in both the IG and CG. Results were summarized by numbers and percentages and compared by chi-squared analysis with a two-tailed criterion (α < 0.05), using the identical cut-off scores for PSS-10, GAD-7, and PHQ-9 scales, respectively.

To assess the impact of the supportive text message intervention on the clinical measures while controlling for sociodemographic characteristics, we entered all demographic predictors along with the “intervention arm” into three different binary logistic regression models. We examined the odds ratios from the regression models to determine the respective associations between the “intervention arm” and the likelihood of respondents to self-report symptoms of moderate to high stress, likely GAD, and likely MDD, controlling for the other sociodemographic variables in the respective model. Correlation analyses were performed before the regression analysis to rule out very strong correlations (r_s_ ≥ 0.7) among predictor variables.

There was no imputation for missing data, and the total numbers reported represent the total responses recorded for each variable.

## Results

Table 1 demonstrates the distribution of the sociodemographic characteristics against the two study groups (CG and IG). There were 286 male subscribers to the Text4Hope service during the timeframe of the study, with 72 (25.2%) in the CG and 214 (74.8%) in the IG. From the table, the majority of the participants were above 40 years (183, 64.2%), white (237, 83.5%), had postsecondary education (208, 85.2%), employed (163, 67.1%), or in a relationship (154, 62.9%), and owning home (164, 68%). IG subscribers were significantly older and had higher education levels compared to CG.

**Table 1:** Distribution of demographic characteristics and isolation conditions of the participants.

**Variables**	**CG** ***n* = 72**	**IG** ***n* = 214**	**Total** ***n* = 286**	**Chi^2^**	***P*-value**
**Age group**
< =40 y	34 (47.9)	68 (31.8)	102 (35.8)	6.02	0.01
>40 y	37 (52.1)	146 (68.2)	183 (64.2)		
**Ethnicity**
White	64 (90.1)	173 (81.2)	237 (83.5)	3.07	0.08
Non-white	7 (9.9)	40 (18.8)	47 (16.5)		
**Education level**
Postsecondary education	56 (77.8)	152 (88.4)	208 (85.2)	4.53	0.03
High school diploma or less	16 (22.2)	20 (11.6)	36 (14.8)		
**Employment status**
Employed	45 (62.5)	118 (69.0)	163 (67.1)	0.97	0.32
Unemployed	27 (37.5)	53 (31.0)	80 (32.9)		
**Relationship status**
In a relationship	45 (62.5)	109 (63.0)	154 (62.9)		
Not in a relationship	27 (37.5)	64 (37.0)	91 (37.1)	0.01	0.94
**Housing status**
Own a home	45 (64.3)	119 (69.6)	164 (68.0)	0.64	0.42
Other	25 (35.7)	52 (30.4)	77 (32.0)		

### Severity analysis

Table 2 summarizes the results of the difference between the three primary outcome variables, along with the CMH score, between the IG and CG. From the table, the intervention group consistently reported a significantly lower mean score across all outcome variables (*p* < 0.05). The mean scores on the PSS-10, GAD-7, and PHQ-9 scales and the CMH score were higher for the CG compared to the IG, 11.4, 28.8, 25.8, and 18.7%, respectively.

**Table 2:** Independent sample t-test comparing the mean scores for IG and CG on PSS, the GAD-7 and PHQ-9 scales and the Composite Mental Health (CMH) score.

**Measure**	**Scores**				**Mean difference (95% CI)**	***P*-value**	***t*-value (df)**	**Effect size (Hedge's g)**
	***n***	**CG, mean (SD)**	***n***	**IG, mean (SD)**				
PSS-10 total score	67	20.81 (8.25)	200	18.68 (7.33)	2.13 (0.03–4.24)	**0.047**	1.99	0.3
PHQ-9 total score	62	10.95 (6.89)	187	8.5 (6.55)	2.45 (0.54–4.37)	**0.01**	2.52	0.4
GAD-7 total score	60	9.32 (6.09)	185	7.41 (5.36)	1.91 (0.28–3.53)	**0.02**	2.31	0.3
CMH Score	60	41.00 (19.24)	185	34.53 (17.71)	6.47 (1.18–11.77)	**0.02**	2.41	0.4

CI, confidence interval; CMH, Composite Mental Health Score. Bold values are *p*-values that are *p* < 0.05.

### Prevalence analysis

Table 3 demonstrates the difference in the prevalence of moderate to high stress, likely depression, and likely anxiety between the IG and the CG of male subscribers. The IG had significantly lower likely MDD (58.2 vs. 37.4%) and likely GAD (50.0 vs. 30.8%) compared to the CG, with a small effect size.

**Table 3:** Chi-square test of association between prevalence of clinical parameters and study arm.

	**Prevalence n/N (%)**				
**Clinical Condition**	**CG**	**IG**	**χ2 (df)**	***p*-value**	**Effect size**
Moderate-to-High Stress	55/67 (82.1)	148/200 (74.0)	1.80 (1)	0.18	0.08
Likely MDD	36/62 (58.1)	70/187 (37.4)	8.11	**< 0.01**	0.18
Likely GAD	30/60 (50)	57/185 (30.8)	7.29	**< 0.01**	0.17

Bold values are *p*-values that are *p* < 0.05.

### Regression analysis

Table 4 shows the binary logistic regression models examining the effect of the provided intervention on the likelihood of the respondents presenting with moderate to high stress, likely depression, and likely anxiety. There was no high correlation (r_s_ < 0.7) between the suggested factors (sociodemographic and intervention type); thus, all variables were entered into the models.

**Table 4:** Odds for subscribers in the IG to have various clinical characteristics compared to the CG.

**Clinical variables of interest**	***p*-value**	**Odds ratio**	**95% CI for OR**	
			**Lower**	**Upper**
Moderate/High stress^a^	0.19	0.60	0.28	1.29
MDD likely^b^	**0.02**	0.47	0.25	0.89
GAD likely^c^	**0.02**	0.47	0.24	0.90

^a^Moderate or High Stress defined as PSS ≥ 14, ^b^Likely GAD defined as GAD-7 ≥ 10, ^c^Likely MDD defined as PHQ-9 ≥ 10. Bold values are *p*-values that are *p* < 0.05.

From Table 4, the results show that the intervention group was a significant predictor for lower odds of both likely MDD and likely GAD while controlling for the sociodemographic variables (including age and educational status, which showed a significant difference between the CG and IG on chi-square analysis) in the two models.

Regarding the likely MDD, the full model was significant, *x*^2^ (df = 7, *n* = 218) = 19.46, *p* < 0.01, explaining between 8.5% (Cox and Snell *R*^2^) and 11.4% (Nagelkerke *R*^2^) of the variance and correctly classified 63.8% of all cases. Controlling for all demographic characteristics, the “intervention arm” contributed significantly to the model (Wald = 5.36). The IG was 0.47 times less likely to meet the cut-off threshold for likely MDD during the study period compared to CG (OR = 0.47; 95% CI = 0.25–0.89). This suggests that participants in the CG were 2.13 times more likely to meet the cut-off threshold for likely MDD compared to participants in the IG, controlling for other variables in the regression model.

In terms of likely GAD, the model was significant, *x*^2^ (df = 7, *n* = 214) = 17.77, *p* = 0.01, explaining between 8% (Cox and Snell R2) and 10.9% (Nagelkerke R2) of the variance and correctly classified 71% of all cases. Controlling for all demographic characteristics, the ”intervention arm“ contributed significantly to the model (Wald = 5.12). The IG was 0.47 times less likely to present with likely GAD during the study period compared to CG (OR = 0.47; 95% CI = 0.24–0.90). This suggests that participants in the CG were 2.13 times more likely to meet the cut-off threshold for likely GAD compared to participants in the IG, controlling for other variables in the regression model.

Regarding stress symptoms, the full model was significant, *x*^2^ (df = 7, *n* = 228) = 26.32, *p* < 0.001, explaining between 10.9% (Cox and Snell R2) and 16.3% (Nagelkerke R2) of the variance and correctly classified 75.4% of all cases. Controlling for all demographic characteristics, the ”intervention arm" failed to predict the likelihood of reporting stress symptoms among the respondents. i.e., respondents in the IG were not significantly more or less likely to experience stress symptoms during the study period compared to the CG (OR = 0.6; 95% CI = 0.28–1.29).

## Discussion

This study was a naturalistic randomized controlled trial that examined the effectiveness of Text4Hope among two groups of male subscribers. Our results suggest that Text4Hope helped to support the mental health wellbeing of male participants who received the texts compared to those who did not. Significant differences were observed in favor of the subscribers who received the text messages for 6 weeks. A significant difference was noted in the severity and the prevalence of GAD and MDD scores; however, regarding stress symptoms, the improvement was reported only for the prevalence but not for the severity of the condition. Furthermore, the intervention group was a significant predictor of lower odds for both likely MDD and likely GAD.

The study results have partially met the predetermined hypothesis, where symptom severity of both anxiety and depression have reached and exceeded the study target of a 25% difference between the two study groups. Similarly, the prevalence of the two conditions (likely GAD and likely MDD) was significantly lower in the IG compared to the CG, albeit the difference was only 20%.

Texting services generally have several advantages; they do not compromise physical distancing requirements while allowing users to receive essential mental health support (18). Moreover, the service is scalable, convenient, and economically reliable, with growing evidence of its applicability in mental health (29). This study's findings, therefore, are consistent with the recently reported results from the Text4Hope service (23, 30). More than 30,000 subscribers joined the service in the first week of its launch (20). After 6 weeks of receiving the daily text message, the service's subscribers reported remarkable satisfaction and acceptability of the service, with a general agreement that the service improved their quality of life and to cope with stress, anxiety and depression (30). Furthermore, more than 4 in five subscribers felt that they are connected to a support system during the pandemic (30). In clinical terms, Text4Hope has significantly improved the baseline mental health symptoms among service subscribers, represented in stress symptoms, likely anxiety, and likely depression; where the positive impact was reported on the short and long-term of the service, i.e., after receiving the daily text messages for 6 weeks (mid-point) and 3 months (end of the service) (22, 23).

Being in the IG had a significant effect on predicting future mental health status. It was a protective factor against the symptoms of depression and symptoms of anxiety, regardless of the baseline sociodemographic characteristics of the participants. Again, this highlights the value of the provided service, where after 6 weeks, it could protect the mental wellbeing of male subscribers. In consideration of the fact that supportive text messages are perceived as add-on services and not meant in any way to replace the conventional lines of therapy, the effect of most psychiatric interventions is mainly achieved within or after the first 6 weeks of treatment (31–34). Further, the assessment of the change in the symptoms is often recommended after the lapse of this time period (34, 35). Considering this, texting service is not outside the scope of other helplines and may be comparable with the effect of these interventions in terms of their effectiveness and timeline benefits.

The present study pointed out the effect of Text4Hope among male subscribers of the service, contradicting the widely accepted notion that any detected effectiveness of such online services is invariably linked to most female participants and not likely to be generalized to the male minority. This follows the fact that most subscribers of such services are typically females, while males represent one in five at most (17–19). Such effectiveness could highlight the need for similar help channels and public health support for the male equal to the female population.

Finally, it is important to mention that in contrast to the anxiety and depression symptoms, stress symptoms seemed quite resistant to handle with Text4Hope among the male participants. As mentioned earlier, emotional stress is a prominent problem in men that could manifest in diverse ways, even different from women's presenting symptoms. This may explain why the texting services are well-accepted, though the male subscribers seemed less satisfied than their female counterparts (30). Collectively, this may raise the urgent need to carefully address such symptoms among men, particularly during crisis times, such as the current pandemic, aiming to guarantee satisfaction and the good uptake of the service.

This study has several limitations. The small sample size of male subscribers might limit the power of the produced results, which may necessitate further research on a larger study sample of the male population. Additionally, the study followed a naturalistic design that lacked the classic randomization of the participants into the two study groups. An effort was made, however, to control for potential differences based on sociodemographic factors using the logistic regression analysis. Finally, the Text4Hope service couldn't manage the stress symptoms as effectively as it did with the depression and anxiety symptoms, which may necessitate further work to address stress, particularly the emotional stress among males if aiming to support this vulnerable population.

To conclude, Text4Hope is an effective service for mental health support for the male population. Males who received the service for 6 weeks were in better mental health status compared to those who didn't, particularly regarding depression and anxiety symptoms. Further effort is still needed to encourage males to participate in such services that can help them receive effective support, particularly during crisis times. More importantly, it is imperative to establish effective lines of help and support tailored to the male population through a collaborative effort of community partners and stakeholders. The online support services, such as Text4Hope, would therefore need extra work to acknowledge the male population while designing the service. Such efforts should prioritize the involvement of male representatives who can effectively contribute while building supportive programs, thus guaranteeing that the peculiar mental health needs related to this neglected population are met.

## Data availability statement

The raw, de-identified data supporting the conclusions of this article will be made available by the authors without undue reservation.

## Ethics statement

The studies involving human participants were reviewed and approved by the University of Alberta Health Research Ethics Board (protocol code Pro00086163 approved on March 18, 2020). Written informed consent for participation was not required for this study as approved by the University of Alberta Health Research Ethics Board.

## Author contributions

Conceptualization, funding acquisition, investigation, project administration, and supervision: VA. Data curation: RS, WV, AGu, SS, and VA. Formal analysis and methodology: VA and RS. Writing—original draft: RS. Writing—review and editing: RS, BA, WV, MH, AGu, SS, AGr, and VA. All authors contributed to the article and approved the submitted version.

## Funding

This study was supported by grants from the Mental Health Foundation, the Calgary Health Trust, the University Hospital Foundation, the Alberta Children's Hospital Foundation, the Royal Alexandra Hospital Foundation, the Alberta Cancer Foundation, Alberta Health Services, and the University of Alberta. The funder had no role in the design and conduct of the study, collection, management, analysis, interpretation of the data; preparation, review, and approval of the manuscript; or the decision to submit the results for publication.

## Conflict of interest

The authors declare that the research was conducted in the absence of any commercial or financial relationships that could be construed as a potential conflict of interest.

## Publisher's note

All claims expressed in this article are solely those of the authors and do not necessarily represent those of their affiliated organizations, or those of the publisher, the editors and the reviewers. Any product that may be evaluated in this article, or claim that may be made by its manufacturer, is not guaranteed or endorsed by the publisher.
